# Microencapsulated Spent Coffee Grounds Extracts Inhibit Enzymatic Browning In Vitro and In Silico

**DOI:** 10.3390/foods15172992

**Published:** 2026-08-26

**Authors:** Nonhlanhla Mathanga Sibisi, Lusani Norah Vhangani, Adane Tilahun Getachew, Charlotte Jacobsen, Tumisi Beiri Jeremiah Molelekoa

**Affiliations:** 1 Center for Innovative Food Research, Department of Biotechnology and Food Technology, Faculty of Science, University of Johannesburg, Doornfontein, Johannesburg 2028, Gauteng, South Africa; nonhlanhlasibisi74@gmail.com; 2 Department of Food Science & Technology, Cape Peninsula University of Technology, Bellville, Cape Town 7535, Western Cape, South Africa; vhanganil@cput.ac.za; 3 Research Group for Bioactives–Analysis and Application, National Food Institute, Technical University of Denmark, 2800 Lyngby, Denmark; atige@food.dtu.dk (A.T.G.); chja@food.dtu.dk (C.J.)

**Keywords:** spent coffee grounds (SCG), apple juice, microencapsulation, enzymatic browning, molecular docking

## Abstract

Enzymatic browning, a reaction catalyzed by polyphenol oxidase (PPO), occurs when polyphenolic compounds in freshly cut produce are exposed to oxygen, reducing sensory quality and consumer acceptability. This study investigated the use of microencapsulated spent coffee grounds (SCG) as a natural inhibitor of browning in 100% apple juice. Phytochemicals were extracted using ultrasound-assisted solvent extraction and subsequently microencapsulated via freeze-drying with maltodextrin, gum arabic, and their combination (1:1) as carrier agents. The encapsulated extracts were evaluated for phenolic and flavonoid content, antioxidant capacity, and anti-browning activity using colorimetric analysis and molecular docking. At the same time, structural characteristics were confirmed using scanning electron microscopy (SEM), Fourier transform infrared spectroscopy (FTIR), and encapsulation efficiency assessment. The results indicated successful encapsulation, with efficiencies exceeding 70%, and morphology and spectra image confirmed the incorporation of phenolic compounds. Amongst the six identified phenolic compounds, trans-ferulic acid was identified as the predominant phenolic compound (14.50 mg/g), while p-coumaric acid was present at lower levels (0.60 mg/g). Application of microcapsules in apple juice significantly reduced the browning index (29.91, 22.43, and 21.48) and increased lightness (78.63, 82.24, and 81.42) over a five-day storage period, demonstrating potential anti-browning of activity. Molecular docking further revealed that flavonoids such as quercetin, luteolin, and apigenin exhibited strong binding affinity for PPO, suggesting their potential as enzymatic inhibitors. Overall, the findings provide primary evidence for the potential use of SCG-derived polyphenols as sustainable, natural antioxidants, contributing to agro-waste valorization.

## 1. Introduction

Enzymatic browning is a chemical reaction prevalent in fruits, vegetables, and their by-products. While it can be beneficial in certain food products, it can also be undesirable. For example, in products such as coffee and tea, this reaction is desired and part of processing, whereas in fruits, vegetables, and their byproducts, it has a negative impact [1]. This reaction alters the texture, quality, and nutritional properties of fresh produce. Consequently, it reduces consumer acceptability of these products and contributes to food waste throughout the food supply chain. The undesired browning reaction is catalysed by the endogenous enzyme polyphenol oxidase (PPO) in the presence of oxygen. This reaction results in the formation of quinones that polymerize into the brown pigment melanin after injury or mechanical damage, during post-harvest, and processing [2].

Apple juice is a fresh product susceptible to enzymatic browning. This reaction affects the consumer acceptability of fruit juice by negatively impacting its taste, colour, and nutrient profile. According to Nehme et al. [3], it is one of the most consumed fruit beverages globally, enjoyed by people of all ages for its authentic flavour. Besides its flavour, apple juice is appreciated for its nutritional benefits, including those from phenolic compounds with anti-inflammatory and neuroprotective properties [3]. Thus, the food industry employs various methods to mitigate or slow enzymatic browning.

The food sector uses several approaches to prevent enzymatic browning. These methods include physical and chemical treatments, although each has its own limitations. Physical methods used to mitigate browning in food include refrigeration, thermal treatments, and filtration. Thermal treatments deactivate enzymes, thereby inhibiting browning [2]. However, they reduce the nutrients inherent in apples because some are heat sensitive. Refrigeration slows the reaction for a limited time, but it is not effective in the long term [4]. Filtration removes unwanted enzymes that catalyse the browning reaction. However, its disadvantages include limited applicability to liquid products, the potential to filter out other important nutrients, such as polyphenols, flavonoids, and other bioactive compounds, and high cost [5]. The food industry has developed alternative non-thermal processes that inhibit browning, including ohmic heating, irradiation, pulsed electric field, and ultraviolet treatment, which are expensive, energy-intensive, and, to some extent, degrade food quality [6].

Chemical methods used in the food sector to combat enzymatic browning are classified by their mechanism. They include antioxidants, acid regulators, chelating agents, and reducing agents that reduce the o-quinones formed in the browning reaction back to phenols or deactivate polyphenol oxidase (PPO). In contrast, antioxidants react with quinones, preventing the formation of the brown pigment melanin [7]. Chelating agents, for example, react with copper at the active site of the catalyzing enzyme to prevent browning. Acid regulators lower the pH below the point at which PPO activity is completely altered [8]. These chemicals are often preferred for their performance and cost-effectiveness. However, most synthetic chemicals are subject to regulations due to safety concerns. Their effectiveness depends on the concentration applied to the food product. There is also a growing trend for clean-label products. For example, regulatory bodies have investigated sulfites for their potential to trigger allergic reactions [9]. For this reason, other alternative sources of antioxidants can be developed to address the challenges mentioned above.

Coffee is an agricultural product commonly consumed as a hot beverage worldwide and is one of the most traded commodities, ranking just after petroleum and its derivatives [10]. Research indicates that approximately 500 billion cups of coffee are consumed worldwide each day [11]. Due to high market demand, a large amount of waste is generated during coffee production, including pulp, silver skin, parchment, and spent coffee grounds (SCG) [12]. SCG are the water-insoluble parts of coffee beans that precipitate at the bottom after brewing. They are typically discarded as waste and end up in landfills [13]. Through decomposition, carbon dioxide and methane are released, contributing to the global warming crisis. However, studies demonstrate that SCG contain valuable nutrients, including phytochemicals, proteins, and polysaccharides such as cellulose, fatty acids, and lignin [13,14,15]. Therefore, SCG are significant in the food industry because they can be used to extract constituents that can be further utilized for various applications, ensuring sustainability.

Since phenolic compounds are adversely affected by environmental factors such as heat, oxygen, and light, and have a bitter taste, it is important to use a method that mitigates these disadvantages. Microencapsulation by freeze-drying, a potential technique to mitigate these disadvantages, uses a wall material that entraps core molecules and forms a permeable layer [16]. Freeze-drying involves freezing the product, followed by reducing the surrounding pressure to allow ice to sublime within the material [17]. The process can prevent degradation of heat-sensitive materials and thus can be used for phytochemicals, proteins, or enzymes; however, it has a high capital cost [18]. Commonly used polymers for microencapsulation include gum arabic (GA), whey protein, and maltodextrin (MA). Carrier systems can directly benefit the beverage sector, particularly fruit drinks.

Spent coffee grounds generate significant waste that ends up in landfill sites and contributes to global warming. On the other hand, apple juice is a food product enjoyed for its taste and flavour, as well as the added nutritional benefits it offers. However, it is susceptible to enzymatic browning. Current methods used to mitigate browning have safety concerns, are subject to regulation, and degrade nutrient quality. Therefore, microencapsulation of the SCG extract provides a promising approach to stabilize, protect, and facilitate its application in apple juice to inhibit enzymatic browning. There is limited research that highlights the use of microencapsulated SCG extract as a natural antioxidant to prevent enzymatic browning in fruit drinks. Previous studies have investigated the effects of freeze-drying versus spray-drying for microencapsulating SCG extract with maltodextrin and gum arabic, evaluated the use of *Saccharomyces cerevisiae* cells to microencapsulate SCG bioactive compounds and ensure their stabilization during digestion, and used coffee byproducts (husks) to inhibit lipid and protein oxidation in beef patties [15,19,20]. However, a research gap remains regarding how wall materials, such as gum arabic and maltodextrin, preserve phenolic compounds and antioxidant capacity in food products, such as fruit drinks. This study addresses this gap by investigating the physical and chemical inhibitory effects of microencapsulated SCG extract on browning in apple juice. It further expands on the use of molecular docking as an in silico technique to measure the binding affinity of selected phenolic compounds with PPO in apple juice. The study hypothesis is that the encapsulated SCG extract will inhibit enzymatic browning and that molecular binding analysis will correlate with this inhibition.

## 2. Materials and Methods

All chemicals and reagents were of analytical grade and purchased from Sigma-Aldrich (Johannesburg, South Africa). Apples were purchased from Checkers Hyper (Johannesburg, South Africa), while spent coffee grounds (SCG) were sourced from local coffee shops (Johannesburg, South Africa).

Chemicals and reagents included methanol, hydrochloric acid, acetonitrile, sodium hydroxide, sodium nitrite, sodium carbonate, DPPH, Folin–Ciocalteu reagent, ABTS, potassium persulfate, and aluminum chloride. The standards included sinapic acid, p-coumaric acid, luteolin, quercetin, apigenin, trans-ferulic acid, and gallic acid. All chemicals, standards and reagents were of analytical grade and purchased from Sigma-Aldrich (Johannesburg, South Africa).

### 2.1. Sample Preparation and Extraction

The extraction was completed, as previously reported by Okur et al. [21], with minor modifications. Spent coffee grounds (from Arabica coffee beans) sourced from local coffee shops were frozen at −20 °C (Defy, Durban, South Africa) and freeze-dried at 0.05 bar and −28 °C for 48 h (Harvest Right, Salt Lake City, UT, USA). The sample was then crushed into fine powder using a mortar and pestle and stored in ziplock bags at 25 °C in a dark room until further use. An acidified 80% methanol solution (pH ~ 3) was prepared by adding 1% HCl to 80% methanol. Spent coffee grounds (0.25 g) were mixed with 5 mL of acidified methanol solution, vortexed for 1 min, and placed in an ultrasonic bath (AU 220, Argo Lab, Carpi, Italy) containing ice water; the temperature was monitored with a thermometer for 1 h at 4 °C. The sample was centrifuged (Eppendorf 5702R, Hamburg, Germany) for 10 min at 2486× *g*. The supernatant was concentrated (Eppendorf, Hamburg, Germany) for 7 h at 30 °C and reconstituted with 2 mL of methanol. This extract was used for subsequent analysis. The extract used for application was reconstituted with 2 mL of 60% ethanol and stored in a refrigerator.

### 2.2. Total Phenolic Compounds (TPC)

A method adapted from Zhong et al. [22], with minor modifications, was used to determine the total phenolic compound concentration in SCGs. Spent coffee grounds extract (10 µL), diluted Folin–Ciocalteu reagent (1:15, 50 µL), and 7.5% freshly prepared Na_2_CO_3_ solution (50 µL) were dispensed into a 96-well microplate. A gallic acid standard solution was also prepared in the range of 0 to 0.2 mg/mL and was used to replace the 10 µL extract. The 96-well microplate was covered with foil and incubated at 25 °C for 30 min. The absorbance was measured using a spectrophotometer (Accuris Instruments SmartReader 96, model MR9600, Jersey City, NJ, USA) at 750 nm. A standard curve was then established using the absorbance value obtained from the gallic acid standard. The curve was used to calculate the TPC in the SCG extract. The analysis was performed in triplicate from the same sample, and the concentration was expressed in mg GAE/g.

### 2.3. Total Flavonoid Content (TFC)

The TFC in the SCGs was determined following the method described by Zhong et al. [22] with some adjustments. A solution consisting of 10 µL extract, 30 µL of 2.5% NaNO_2_, 30 µL of 2% NaOH, and 30 µL of 1.25% AlCl_3_ was transferred to a 96-well microplate. A quercetin standard solution (0–0.2 mg/mL) was used to replace 10 µL of extract and construct a standard curve. The 96-well microplate was covered with foil and incubated at 25 °C for 30 min. The absorbance was measured using a spectrophotometer (Accuris Instruments SmartReader MR9600, Jersey City, NJ, USA) at 734 nm. A standard curve was established using the absorbance value obtained from the Quercetin standard. The analysis was completed in triplicate from the same sample. The TFC in the SCG extract was calculated from the absorbance-concentration curve and expressed as mg QE/g.

### 2.4. 2,2-Azinobis-3-ethylbenzothiazoline-6-sulfonic Acid (ABTS)

A method by Ballesteros et al. [19] was followed, with adjustments for the ABTS assay. To prepare the ABTS solution, 19 mg of ABTS was dissolved in 5 mL of distilled water, and then 3.5 mg of potassium persulfate was weighed in 5 mL of distilled water. The solutions were mixed with an additional 5 mL of distilled water. The solution was incubated for 16 h in a dark room at 25 °C. The 1 mL of the incubated solution was then mixed with 60 mL of distilled water. The 20 µL of extract with 200 µL of ABTS solution in a 96-well microplate. The control was prepared by plating 220 µL of ABTS solution. The microplate was incubated for 1 min at room temperature and covered with foil in a dark room. The absorbance was measured at 734 nm using a spectrophotometer (Accuris Instruments SmartReader 96, model MR9600, Jersey City, NJ, USA). The analysis was completed in triplicate from the same sample and the % inhibition was computed using Equation (1):

(1) $$(\%)\;\mathrm{Inhibition}\;=\frac{(\mathrm{Ac}\;-\;A)}{A}\;\times \;100$$

where Ac—blank absorbance and A—sample absorbance.

### 2.5. 2,2-Diphenyl-1-picrylhydrazyl (DPPH)

Similarly, the antioxidant capacity of the SGC extract was determined using the DPPH assay, as described by Ballesteros et al. [20] with some modifications. To prepare the DPPH solution, 4 mg was diluted to 100 mL with methanol, then 20 µL of the extract was mixed with 200 µL of the DPPH solution for the sample and 220 µL for the control in a dark room. The plating was completed in triplicate from the same sample. The microplate was then covered with foil and incubated in a dark room at 25 °C for 30 min. The reading was obtained using a spectrophotometer (Accuris Instruments SmartReader 96, model MR9600, Jersey City, NJ, USA) at 517 nm. The %inhibition was computed using Equation (2).

(2) $$(\%)\;\mathrm{Inhibition}=\frac{(\mathrm{Ac}\;-\;A)}{A}\;\times \;100$$

where Ac—blank absorbance and A—sample absorbance.

### 2.6. Ultra-Performance Liquid Chromatography (UPLC)

Phenolic acids in the SCG extract were quantified by targeted profiling on a UPLC system (Shimadzu Corporation, Kyoto, Japan) using a method outlined by Okur et al. [21], with some adjustments. The phenolic compound standards included sinapic acid, p-coumaric acid, luteolin, quercetin, apigenin and trans-ferulic acid. The extract was passed through a 0.45 μm nylon membrane fiber before analysis. The analysis was completed using a C18 column (2.1 × 100 mm) with a 3 µm particle size (Waters Chromatography Ireland Limited, Dublin, Ireland), and a quaternary pump. A binary mobile phase consisting of solvent A (ultra-pure water) and solvent B (acetonitrile and methanol, 70:30 *v*/*v*) was used for the separation process. The analysis was carried out under a gradient elution mode: 0 min 10% solvent B, 0–0.50 min 10% solvent B, 0.50–7.00 min 90% solvent B, 7.00–8.00 min 100% solvent B, 8.00–14.00 min 100% solvent B, 14.00–16.00 min 60% solvent B, 16.00–17.00 min 30% solvent B, and 17.00–18.00 min 10% solvent B. The flow rate and column temperature were 0.15 mL/min and 40 °C, respectively. The phenolic compounds in the chromatogram were quantified using a standard curve obtained using the standards (LabSolutions software, Shimadzu Corporation, Kyoto, Japan). The scanning range for each of the phenolic compounds was between 220 nm and 550 nm: apigenin 336 nm, luteolin 348 nm, sinapic acid 319 nm, quercetin 371 nm, p-coumaric acid 309 nm, and trans ferulic acid 322 nm.

### 2.7. Microencapsulation

The microencapsulation of the extract was performed as described by Chacon-Figueroa et al. [20]. The extract was first concentrated for 8 h in an evaporator (Eppendorf, Hamburg, Germany) and then reconstituted with 1–2 mL of 60% ethanol. The multiple independently reconstituted batches of extract samples were subsequently combined to a volume of 7 mL of the extract, which was mixed with 1.4 g of gum arabic (GA) or maltodextrin (MA) using a magnetic stirrer (VELP Scientifica MST Magnetic Stirrer, Usmate, (MB), Italy) until complete dispersion was achieved. The SCG microcapsule containing GA and MA was prepared using GA and MA at a 1:1 ratio. The samples were pre-frozen overnight and freeze-dried (Harvest Right, Salt Lake City, UT, USA) for approximately 48 h at −28 °C. The samples were crushed into a fine powder using a pestle and mortar and stored in amber vials at 4 °C until further use.

### 2.8. % Encapsulation Efficiency (% EE)

The Encapsulation Efficiency (%EE) was calculated as described by Dadi et al. [21], with adjustments. To extract surface phenolic content (SPC) from the coated samples, 0.25 g of each sample was mixed with 5 mL of 80% ethanol. To extract phenolic compounds from the microcapsule, the procedure in Section 2.1 was carried out. Followed by the determination of surface phenolic compounds (SPC) and TPC of the microcapsule using the TPC procedure as described in Section 2.3. The analysis was carried out in triplicate from the same microcapsule batch. The % EE was calculated using Equation (3):

(3) $$\mathrm{EE}\;(\%)\;=\frac{(\mathrm{TPC}\;-\;\mathrm{SPC})}{\mathrm{TPC}}\;\times \;100$$

where TPC is total phenolic content and SPC is surface phenolic content.

### 2.9. Scanning Electron Microscopy (SEM)

The SEM images were obtained using a method outlined by Ballesteros et al. [19] with some adjustments. The microencapsulated samples were placed in aluminum stubs and coated with carbon. The samples were then analysed using Scanning Electron Microscopy (Vega XMU, TESCAN VEGA, Brno, Czech Republic) at 20 kV and a magnification of 1.00 KX.

### 2.10. Fourier Transform Infrared (FTIR)

The FTIR was conducted as outlined by Chacon-Figueroa et al. [18], with modifications. The PerkinElmer Spectrum Two FTIR spectrometer and PerkinElmer software (Waltham, MA, USA) were used to study the functional groups present in (SCG, SCG + MA, SCG + GA and SCG + GA + MA) at a wavelength between 400 and 4000 cm^−1^.

### 2.11. Apple Juice Preparation

The apple juice was prepared using (1.5 kg × 2) Golden Delicious apples purchased from Checkers Hyper (Johannesburg, South Africa). The apples were washed with cold water, peeled, and cut into small pieces. The feed material was then placed in a juicer (Milex Power juicer, Johannesburg, South Africa), after which 0.1% potassium sorbate was added to inhibit microbial proliferation. The juice was then stored at 4 °C until further use.

### 2.12. Application

The positive control was prepared by mixing 1% ascorbic acid with 50 mL of apple juice. The first experimental group consisted of apple juice mixed with a 1% microencapsulated maltodextrin extract. The second experimental group received apple juice and a 1% gum arabic microencapsulated extract. Lastly, the third experimental group consisted of 1% microencapsulated maltodextrin + gum arabic. The negative control was the freshly pressed apple juice. The samples were stored at room temperature for 5 days, with colour changes monitored using various analytical tests.

### 2.13. Colorimetric Analysis

The juice samples (positive control, negative control, and 3 samples) were analysed for colour change using a colorimeter under uniform lighting conditions over 5 days. The colorimeter (Konita Minolta Inc., Tokyo, Japan) was first connected to the data processor (Konita Minolta Inc., Tokyo, Japan) and calibrated using the white calibration plate (y-85.0, x-0.3165, y-0.3231). The juice samples were poured into translucent Petri dishes. The analysis was performed in triplicate from the same batch of juice samples. The colorimeter head was placed on the empty Petri dish to measure its colour using the LAB Colour space, followed by measuring the colour of the juice samples using the CIELAB colour space. The L* (lightness), a* (red, green), and b* (yellow, blue) colour indices were measured and recorded. The values obtained were used to compute the browning index (BI) using Equations (3) and (4), with the coefficients adapted from Jan et al. [22].

(4) $$\mathrm{BI}\;=\;\frac{x\;-\;0.31}{0.172\;}\times \;100$$

where $x=\frac{a*\;+\;1.75\;L*}{5.645\;L*\;+\;a*\;-\;3.012\;b*}$.

### 2.14. Molecular Docking Between the Polyphenolic Oxidase and Polyphenols

#### 2.14.1. Preparation of the Enzyme and Receptor

Molecular docking was performed according to the methodology outlined by Dilek [15]. The 3D structure of the receptor MdPPO1 was obtained from the Protein Data Bank (PDB ID: 6ELT) and saved as a PDB file. The 3D structure of the ligands quercetin, luteolin, apigenin, p-coumaric acid, sinapic acid, and trans-ferulic acid was obtained from PubChem and saved as SDF files. The protein 3D structure was first minimized using UCSF Chimera (Version 1.17.3, San Francisco, CA, USA).

#### 2.14.2. Molecular Docking Analysis

Molecular docking was performed using AutoDock Vina 1.1.2 (Win32), operated with UCSF Chimera (Version 1.17.3, San Francisco, CA, USA). The binding site for the MdPPO1 enzyme was defined using a grid box covering the entire 3D protein structure, with an approximate grid size of 62.82 × 33.94 × 77.63 Å and a center at x = 15.71, y = 39.03, and z = 75.35. The AutoDock Tool was used before docking to remove nonpolar hydrogens and lone pairs, merge charges, and add hydrogens to the receptor. The ligand’s charges were merged, and non-polar hydrogens and lone pairs were removed.

#### 2.14.3. Post-Molecular Docking Analysis

The enzyme-ligand interaction was analysed in 3D using BIOVIA Discovery Studio (BIOVIA, Dassault Systèmes, Waltham, MA, USA). The software was used to visualize the interactions between each polyphenol and the enzyme.

### 2.15. Statistical Analysis

The data analysis was performed using IBM SPSS Statistics (Version 30; IBM Company, Chicago, IL, USA). It was used to perform a one-way ANOVA. Duncan’s multiple tests were used to differentiate between means at a 95% confidence level (*p* < 0.05) for assays. The FTIR graph was plotted using OriginLab 2025b (OriginLab, Northampton, MA, USA). The reported triplicates in the study represent technical replicates.

## 3. Results and Discussion

### 3.1. Physicochemical Profile of SCG and SCG Conduits in Polymeric Materials

The results in Table 1 showed that the SCG extract had the highest TPC and TFC and the highest % inhibition against DPPH and ABTS compared with the microencapsulated samples. This is because, in microencapsulated samples, the wall material’s mass dilutes the concentration of polyphenols within the microcapsules. Additionally, microencapsulation can facilitate intermolecular bonding between the extract and the polymer [23]. This can contribute to the slow release and entrapment of polyphenols, resulting in their low detection during analysis [24]. Ballesteros et al. [20] observed the same trend for SCG extract, GA, MA, and their combination. In their study, total phenolic content was 216.37, 173.57, and 145.32 mg GAE/g after freeze-drying, relative to 350.28 mg GAE/g in the original extract, showing the same decrease in total phenolics observed in this study. In practice, this would mean that a high number of microcapsules would need to be incorporated into a food product to achieve the same dosage effect as a crude extract. However, this could be a fair trade-off, as microencapsulation would protect polyphenols and ensure their bioavailability for an extended period. In contrast, applying the crude extract directly could be effective immediately but would not be guaranteed over the long term. The extract would be directly exposed to environmental conditions that degrade its polyphenols. Among the three microencapsulated samples, SCG + MA exhibited the lowest TPC, TFC, and antioxidant capacity in the DPPH and ABTS assays. Gum arabic microcapsules alone and in combination with maltodextrin exhibited high antioxidant capacity, consistent with the analysis reported by Araujo et al. [25]. The coated samples showed high antioxidant capacity in DPPH and ABTS assays when gum arabic was used alone or in combination with maltodextrin. This is because gum arabic has a larger particle size than maltodextrin, which enhances the retention of phenolic compounds [26]. Since TPC values are correlated with antioxidant capacity, this may explain the similar trends observed with DPPH and ABTS, with the maltodextrin-coated sample exhibiting the lowest antioxidant capacity compared with gum arabic alone or in combination. Moreover, gum arabic exhibited improved retention of bioactive compounds, whether alone or in combination with maltodextrin, owing to its structure. Gum arabic contains highly branched chains of sugar units, with small proportions of proteins covalently bonded to the carbohydrate backbone, which enable the formation of a stable film and, consequently, the retention of high TPC, TFC, and antioxidant levels [27].

Table 1 illustrates the phytochemical profile of the SCG extract and microencapsulated samples.

### 3.2. Target Phenolic Acid and Flavonoid Composition of SCG Extracts

SCG phenolic acids were quantified using a UPLC instrument. Table 2 shows that the main compound with the highest concentration is trans-ferulic acid (14.50 mg/g), followed by sinapic acid (4.24 mg/g). In contrast, quercetin, apigenin, luteolin, and p-coumaric acids were found in low amounts. Phytochemicals present in SCG have been linked to the natural synthesis of secondary metabolites in coffee plants under stressful conditions [20]. In this study, trans-ferulic acid, an isomer of ferulic acid, was identified as the most abundant phenolic acid among those quantified. Trans-ferulic acid is a phenolic compound known for its high natural antioxidant capacity and its ability to scavenge free radicals [28]. Therefore, this study indicates that trans-ferulic acid is a significant contributor to the antioxidant capacity of the SCG extract and the potential anti-browning activity of apple juice. Ramon-Goncalveds et al. [29] reported trans-ferulic acid concentrations ranging from 0.089 to 0.16 mg/g across different coffee cultivars in SCG, which are lower than the values obtained in this study. Additionally, Vu et al. [30] found ferulic acid concentrations in SCG extract ranging from 40.5 to 1041.7 µg/g of dry weight, making it the second-highest quantified compound, following chlorogenic acid in their study. The second highest concentration quantified in this study was sinapic acid. Badr et al. [31] and Hussein et al. [32] also quantified sinapic acid in SCG extract at concentrations of 17.05 µg/g and 10.10 µg/g, respectively. In the literature, p-coumaric acid in SCG ranged from 0.173 to 0.50 mg/g, while quercetin was not detected [29]. In another study, enzymes were used to extract phytochemicals from SCG over different periods. The concentrations of quercetin and p-coumaric acid ranged around 2.04–6.45 µg/100 g and 7.97–13.93 µg/100 g, respectively [33]. According to Dilek [15], the flavonoids apigenin and luteolin were not detected in the SCG extract because their limits of detection exceeded the concentrations present in the extract. However, in this study, apigenin and luteolin were detected and quantified, indicating their presence in the coffee extract, although in low amounts. The presence of these phytochemicals explains the high % antioxidant capacity and the TPC and TFC values obtained for both the extract and microencapsulated samples in Table 1. Published articles also report varying levels of phytochemicals in SCG extract. This is attributed to differences in extraction methods, solvent-to-sample ratio, extraction solvent, and SCG sources (Arabica or Robusta) [30]. Lastly, the presence of phenolic compounds in SCG, as demonstrated in this study, suggests further applications in the pharmaceutical, food, and cosmetic sectors. In this study, conventional solvents were used to extract these phytochemicals from spent coffee grounds, raising environmental and safety concerns. Future research can explore green extraction solvents to reduce toxicity and improve the environmental sustainability of SCG valorisation.

Table 2 presents the concentrations of targeted phenolic acids and flavonoids in the SCG extract, as quantified by UPLC.

### 3.3. % Encapsulation Efficiency (% EE)

**Figure 1:**
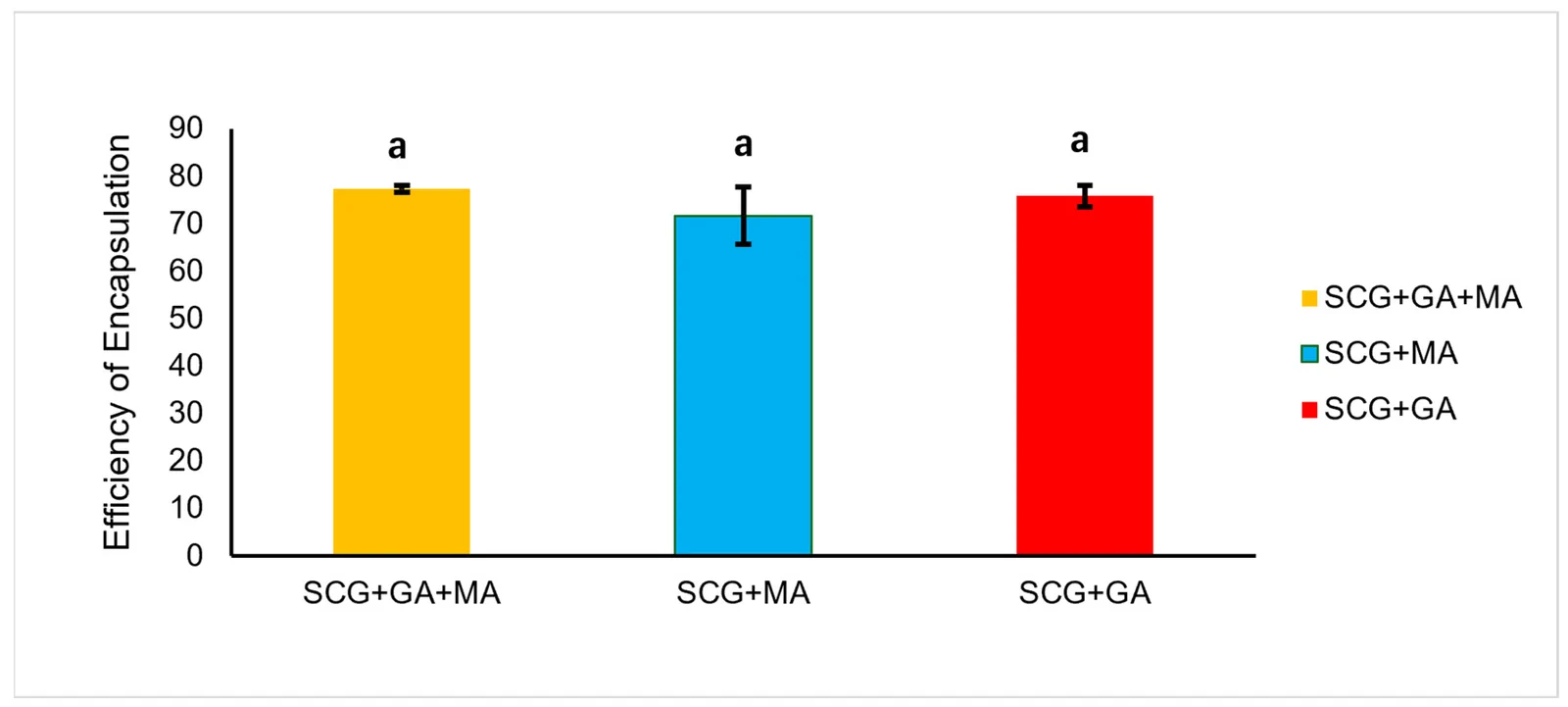
Encapsulation efficiencies of spent coffee ground extract in biopolymers. SCG (spent coffee grounds), MA (maltodextrin), and GA (gum arabic). Values are presented as mean ± standard deviation, where *n* = 3. Bars sharing the same letters are not significantly different from each other (*p* > 0.05).

Figure 1 shows the % EE for microencapsulated and freeze-dried polymers. All three microencapsulated samples exhibited high encapsulation efficiency, ranging from 71.30% to 77.35%. Statistical analysis revealed no significant differences among the microencapsulated samples. This suggests that all carrier wall materials effectively retain phenolic compounds, whether maltodextrin or gum arabic is used individually or in combination. This uniform effectiveness of the carrier walls in encapsulating the SCG extract is attributed to the inherent characteristics of each wall material. The structure of gum arabic, which is highly branched and contains low protein content (arabinogalactan proteins), enables gum arabic to be less viscous in solutions and to have high emulsification and film-forming properties [34]. Furthermore, phenolic compounds can bind to proteins and hydrocarbons through hydrophobic interactions or hydrogen bonding [35]. Maltodextrin is hydrophilic in nature and therefore highly soluble; it has low viscosity, and its ability to complex with different compounds enables it to encapsulate and yield high %EE [36]. This means that maltodextrin and gum arabic have similar capability to achieve higher efficiency in encapsulating phenolic compounds extracted from SCG. In practice, food processors could use maltodextrin or gum arabic, either in combination or individually, based on current market prices and availability, without sacrificing product quality. While microencapsulation by freeze-drying can entail high capital costs in large-scale applications, wider adoption in the food sector over time could reduce these economic barriers.

### 3.4. Microstructural Morphology of SCG Extract Encapsulated in Biopolymeric Materials

**Figure 2:**
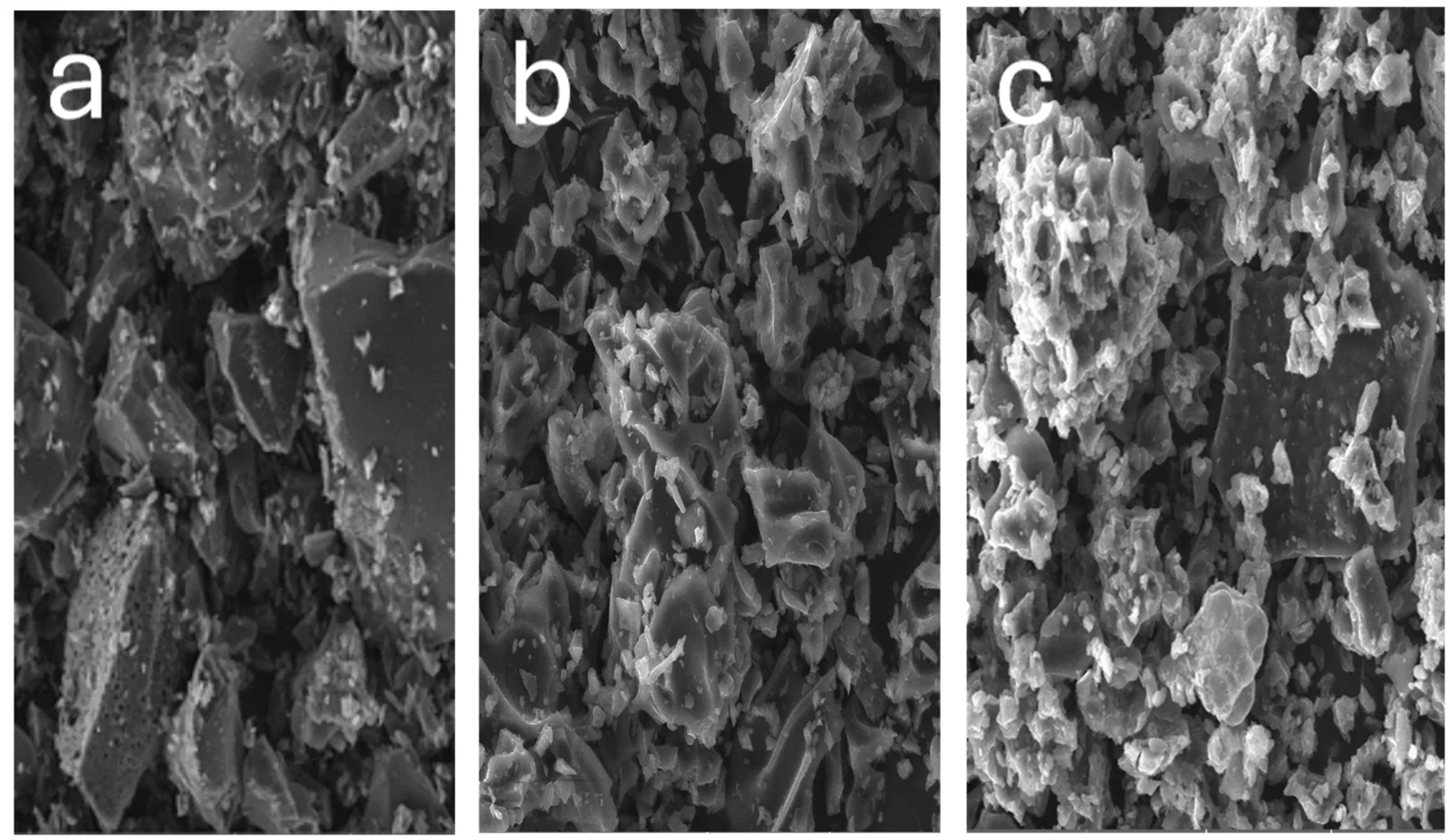
SEM images of microencapsulated samples after freeze-drying. Image (**a**) SCGS + MA + GA, (**b**) SCGS + GA and (**c**) SCGS + MA at 1000× magnification.

The SEM images in Figure 2 show the microstructure of the SCG + MA, SCG + GA, and SCG + MA + GA microcapsules after freeze-drying. Figure 2a depicts particles that are larger and more uniform, with a porous texture and a smoother, less compact surface than in Figure 2c. This suggests that using a combination of gum arabic and maltodextrin retains polyphenols within the capsule, as supported by the high % EE that exceeds 70% observed in Figure 1 when both coating materials were used. Figure 2b depicts aggregated, irregular particles that are smaller than Figure 2a but larger than Figure 2c. This is attributed to the large particle size and hydrophilic, emulsifying properties, which improve protection and encapsulation of the core material [37]. This is evident in the antioxidant capacity results shown in Table 1 for GA, which were higher than those for MA. Figure 2c depicts small, irregular, porous, and broken particles that were agglomerated. Pan-utai and Iamtham [38] also reported these porous, irregular structures for all samples. All images depicted irregular particles. These structures are typically observed during freeze-drying due to sublimation, which alters the microcapsule structure [20]. Furthermore, the variations in particle size observed in images A, B and C can be attributed to the grinding processes applied after freeze-drying the samples [27].

The microstructure of the encapsulated samples obtained through SEM is illustrated in Figure 2.

### 3.5. FTIR Spectral Characteristics of Encapsulated SCG Extracts in Polymeric Material

**Figure 3:**
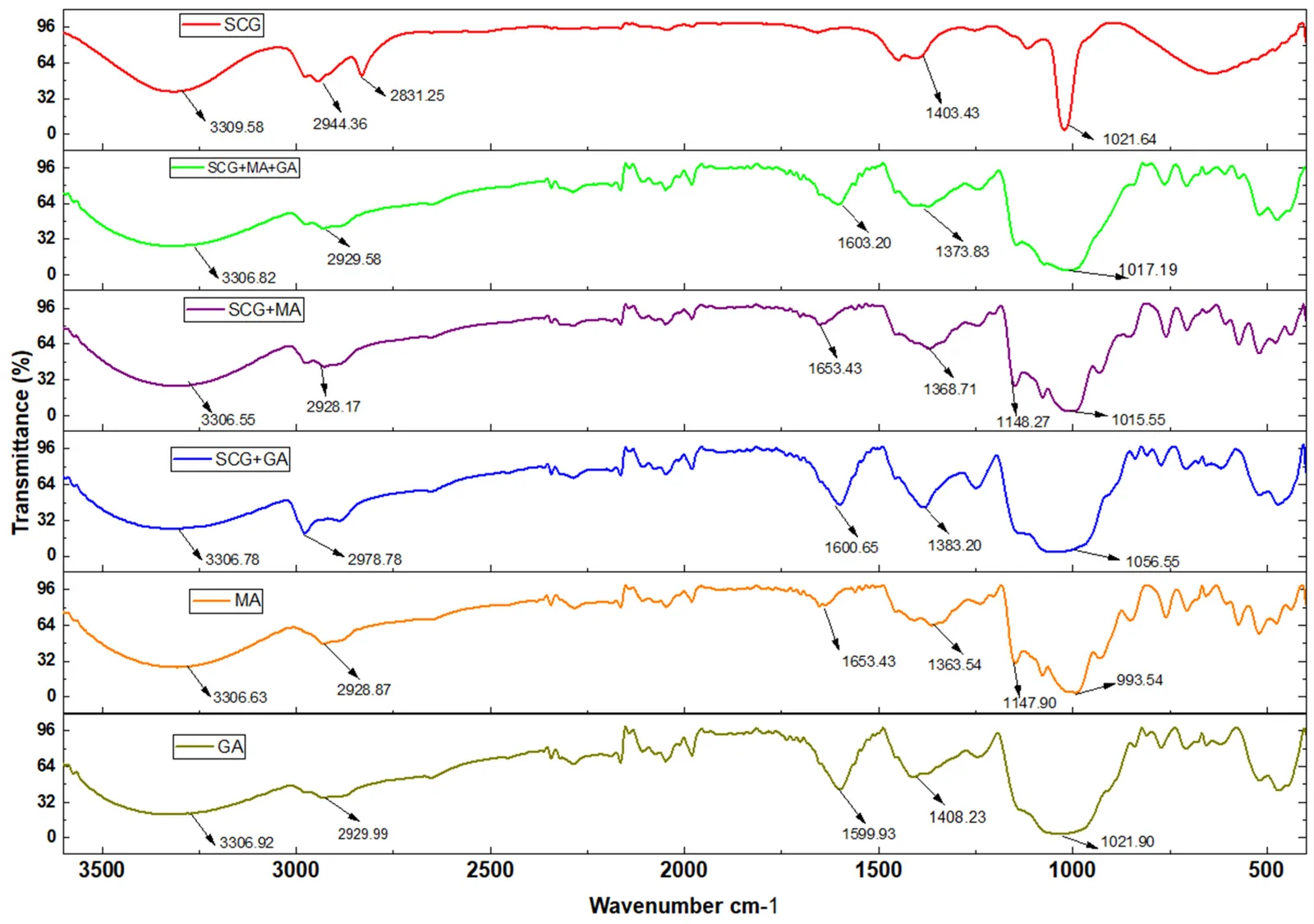
The FTIR results for pure maltodextrin (MA), gum arabic (GA), and the phenolic compounds encapsulated using freeze-drying.

The FTIR spectra for the extract and microencapsulated samples are depicted in Figure 3. The SCG extract depicted a broad band at 3309 cm^−1^, corresponding to the O-H vibration of hydroxyl groups. This confirms the presence of hydroxyl, aromatic, and carbonyl functional groups associated with polyphenols such as p-coumaric acid, caffeic acid, chlorogenic acid, and trans-ferulic acid [20,39]. The presence of trans-ferulic acid and p-coumaric acid was confirmed by targeted profiling, as depicted in Table 2. Peaks at 2944.36 and 2831.25 cm^−1^ were attributed to CH stretching vibrations of aromatic compounds with phenolic bonds, while the band at 1656.02 cm^−1^ represented C=O associated with caffeine and chlorogenic acid. In the region 3600–3000 cm^−1^, the peak at 3306 cm^−1^, often associated with the O-H hydroxyl group of carbohydrates, was observed for all the samples except the SCG extract. A similar peak at 3300 cm^−1^ was observed by Oliyaei et al. [40] for samples coated with gum arabic, maltodextrin and a combination of both. The spectra for gum arabic showed peaks at 2929.87 cm^−1^ (C-H asymmetric), 1599.93 cm^−1^ (amide I band from glycoproteins), and 1408.23 cm^−1^ (amide II band). This indicates peaks associated with glycoprotein and carbohydrate structures that serve as a backbone, as observed by Ballestoeros et al. and Cai et al. [20,41]. On the other hand, the peaks observed in the spectra of maltodextrin, such as hydroxyl and -CH2 stretching vibrations at 2928.87 cm^−1^ and carbonyl groups at 1653.19 cm^−1^, and C-O stretching with C-O-H groups at 1363.54–1077.70 cm^−1^, are associated with the carbohydrate structure [42,43]. The microcapsules (SCG + MA, SCG + GA, SCG + GA + MA) and coating materials (GA and MA) exhibited similar spectra. This indicates that the final sample structures were not really affected by the addition of the SCG extract. However, some peaks in the coated samples shifted slightly compared to the coating materials alone. For example, for GA, there were peak shifts from 2929.99 cm^−1^ to 2978.78 cm^−1^ and from 1408.23 cm^−1^ to 1383.20 cm^−1^ in SCG + GA. In MA, there was a peak shift from 1363.54 cm^−1^ to 1368.71 cm^−1^ in SCG + MA. This suggests minimal interaction between the extract and the coating materials, which is preferred because it helps maintain the structures and bioactivity of the phenolic compounds [44]. Furthermore, slight peak shifts in the coating materials and microencapsulated samples indicate hydrogen-bond interactions between the capsule and core materials, suggesting successful microencapsulation of the SCG extract [45]. Additionally, the peaks observed in GA and MA exhibit a synergistic effect when combined.

The interaction of the chemical bonds of SCG with biopolymers after encapsulation is demonstrated in Figure 3.

### 3.6. Application of Encapsulated SCG in Apple Juice

Table 3 depicts the phenolic content and antioxidant capacity of apple juice added with SCG microcapsules. The positive control sample exhibited high TPC and TFC, and high ABTS % inhibition. This may result from ascorbic acid’s ready availability in the juice and its action as a browning inhibitor via oxygen scavenging, acidulation, or as a reducing agent. Therefore, inhibits the oxidation of available polyphenols, thereby preserving phenolic content and maintaining high antioxidant capacity. The higher TPC and TFC values observed in the positive control could be attributed to the apple juice matrix, including inherent apple juice polyphenols and added ascorbic acid. Ascorbic acid is a strong reducing agent and directly reacts with the Folin–Ciocalteu reagent since it is not specific to phenolic compounds only [46]. This therefore leads to overestimation. In contrast, DPPH % inhibition showed no statistically significant difference between the positive and negative control juice samples. For apple juice samples with added SCG microcapsules, no significant differences (*p* > 0.05) were observed between the encapsulated and negative control for TPC; however, TFC was higher in all microcapsule samples than in the negative control. Microcapsules showed similar TPC and TFC (*p* > 0.05); however, the apple juice: GA sample exhibited the highest % inhibition against DPPH and ABTS radicals. This suggests that GA is more effective in facilitating the rapid release of polyphenols into the juice matrix. Ribeiro et al. (2020) also found that although GA + MA achieved the highest %EE, GA still exhibited rapid polyphenol release [47]. This is attributed to the coating’s solubility, which can influence dissolution and, in turn, affect rapid polyphenol release [48]. This is also evident in the morphology of GA + MA, which showed a dense, continuous layer with larger, smoother particles that can slow polyphenol release compared to GA. However, direct PPO activity was not experimentally measured in the juice samples. Future work can investigate direct inhibition of PPO by the microcapsules to confirm direct inhibition.

Table 3 demonstrates the phytochemical profile of apple juice samples treated with different SCG extracts encapsulated in different polymeric materials.

### 3.7. Colour

**Figure 4:**
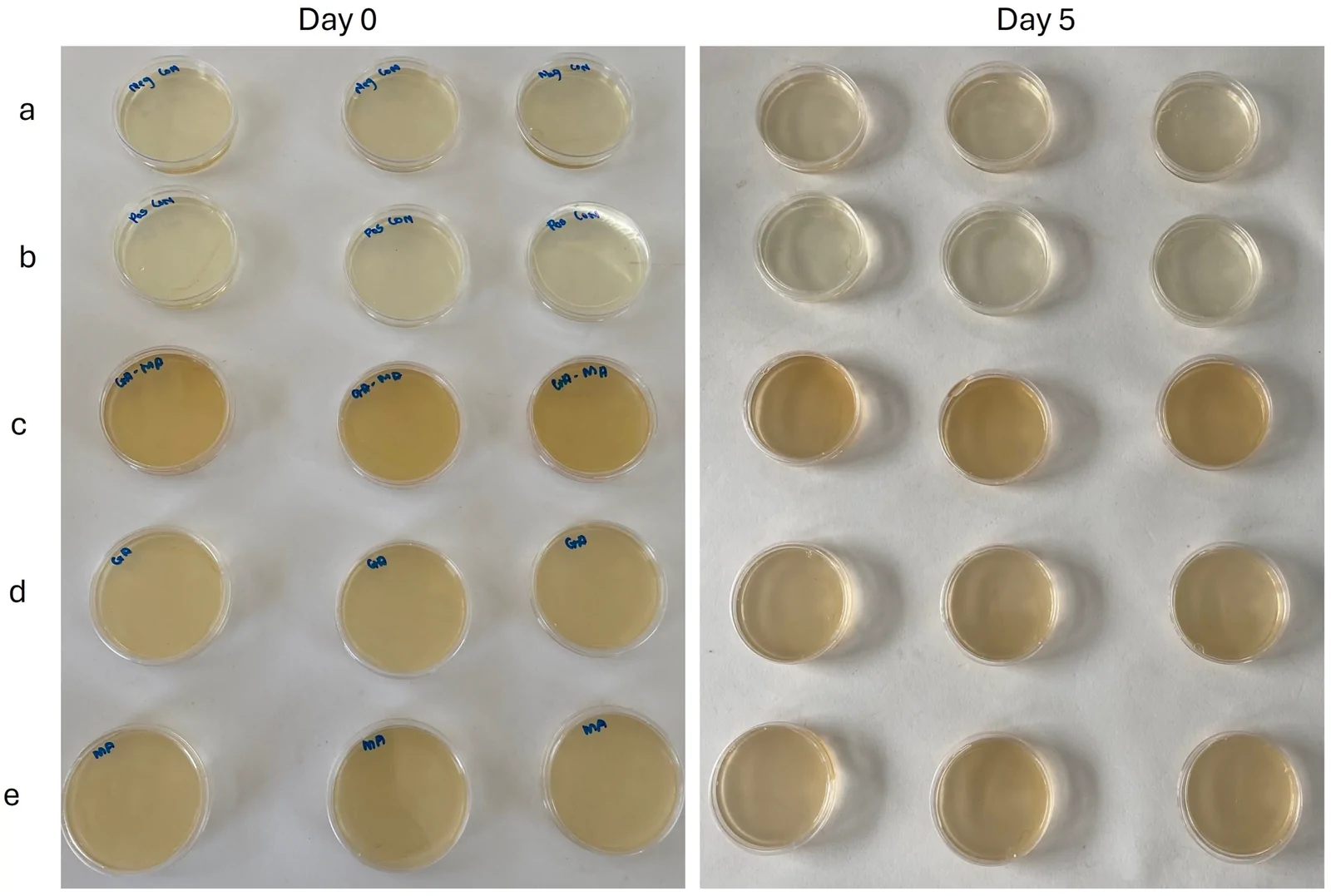
Two images showing the juice samples inside Petri dishes on day 0 (left) and day 5 (right) with (**a**) positive control (apple juice + ascorbic acid) (**b**) negative control (apple juice only), (**c**) (apple juice + (SCG + MA + GA)), (**d**) (apple juice + (SCG + GA)) and (**e**) (apple juice + (SCG + MA)).

Results of colour development via L*, a*, b*, and BI of apple juice with added SCG microcapsules at days 0 and 5 are depicted in Table 4; similarly, Figure 4 shows the juice samples, providing visual confirmation of the results in Table 4. The apple juice samples prepared with the microcapsules were darker brown on days 0 and 5 than the negative and positive controls, owing to the microcapsules’ colour. This dark brown colour is inherent to the SCG crude extract used for microencapsulation. The inherent dark pigmentation of the microcapsules directly influenced the colour calculations, which explains why the treated samples retained higher BI values than the control samples despite the decrease in the BI value. On day 0, all samples had high L* values, with the positive control showing the highest. Among the apple juice samples, the juice prepared with a GA + MA combination microcapsule showed the lowest L* value. This may be attributed to the colour imparted to the juice by the microcapsule during application, as mentioned earlier. The lightness of the positive control increased from 87.46 on day 0 to 88.04 on day 5, thereby decreasing the browning index from day 0 to day 5. Ascorbic acid initially acts as a reducing agent, converting benzoquinones intermediates back to their reduced diphenol form, thereby inhibiting polymerisation and resulting in an increase in lightness (L*) [49]. This action indicates ascorbic acid’s immediate action and how it continued to inhibit browning in the apple juice during the five-day storage. In addition to its reducing ability, ascorbic acid inhibits browning by reacting with PPO, acting as a chelating agent that removes copper ions from the enzyme’s active site [50]. In contrast, the lightness of the negative control remained the same from day 0 to day 5, while its yellowness (b*) and BI increased. This confirms the absence of antioxidants that inhibit browning. For apple juice prepared with microcapsules, the GA + MA combination exhibited the lowest L* values on days 0 and 5 (77.37 and 78.63, respectively), thereby yielding the highest BI on those days. Visually, Figure 4 shows that juice prepared with the GA + MA microcapsule combination was darker than the other samples on both day 0 and day 5. This is because the GA + MA combination leads to high polyphenol retention, which correlates with greater retention of colour compounds [51]. These compounds may have been released into the juice during application, leading to the darker colour. It is also observed that although this juice showed the lowest L* value, it concomitantly had a high BI. Furthermore, the juice sample still showed the largest decrease in BI compared with those prepared with MA and GA, including the positive control. This indicates that antioxidants released by the GA + MA microcapsule into the juice sample may have potentially inhibited browning. Additionally, the denser, more protective wall structure of the GA + MA microcapsule, as evidenced by its morphology, effectively controlled polyphenol release into the juice, thereby protecting the juice from environmental factors. Table 4 also shows that juice samples prepared with MA and GA microcapsules showed potential anti-browning activity in apple juice, as both samples showed a decreased BI on day 5, while there was also a slight increase in lightness.

In practice, this means microcapsules could potentially be used as anti-browning agents in fruit drinks. However, they pose a challenge by imparting a brown colour that consumers may find visually unappealing, especially in apple juice. To overcome this limitation, purified or individual polyphenols isolated from SCG could be encapsulated rather than using a crude extract. Alternatively, microcapsules could be incorporated as antioxidants in food products where colour change would be unnoticeable or where oxidative stability is a major concern. These products include dark sauces (soy sauce or barbecue sauce) and chocolate, or cocoa-based products. Additionally, optimizing the microcapsule dosage may help identify a threshold that effectively inhibits enzymatic browning without changing the colour of the juice. Furthermore, the colour change of the juice was evaluated over 5 days, which may not reflect long-term browning behaviour or stability, thereby indicating a need for prolonged storage analysis, coupled with the study of the release kinetics of encapsulated polyphenols from the wall materials. Another limitation is that the independent effects of the empty carrier wall and crude extract were not evaluated. Consequently, the effect of the crude extract versus the microcapsules cannot be fully isolated.

Figure 4 illustrates the apple juice samples treated with ascorbic acid and encapsulated SCG extracts on days 0 and 5.

Table 4 demonstrates the colour parameters and browning index (BI) of apple juice samples with the microencapsulated samples and ascorbic acid.

### 3.8. Molecular Docking

**Figure 5:**
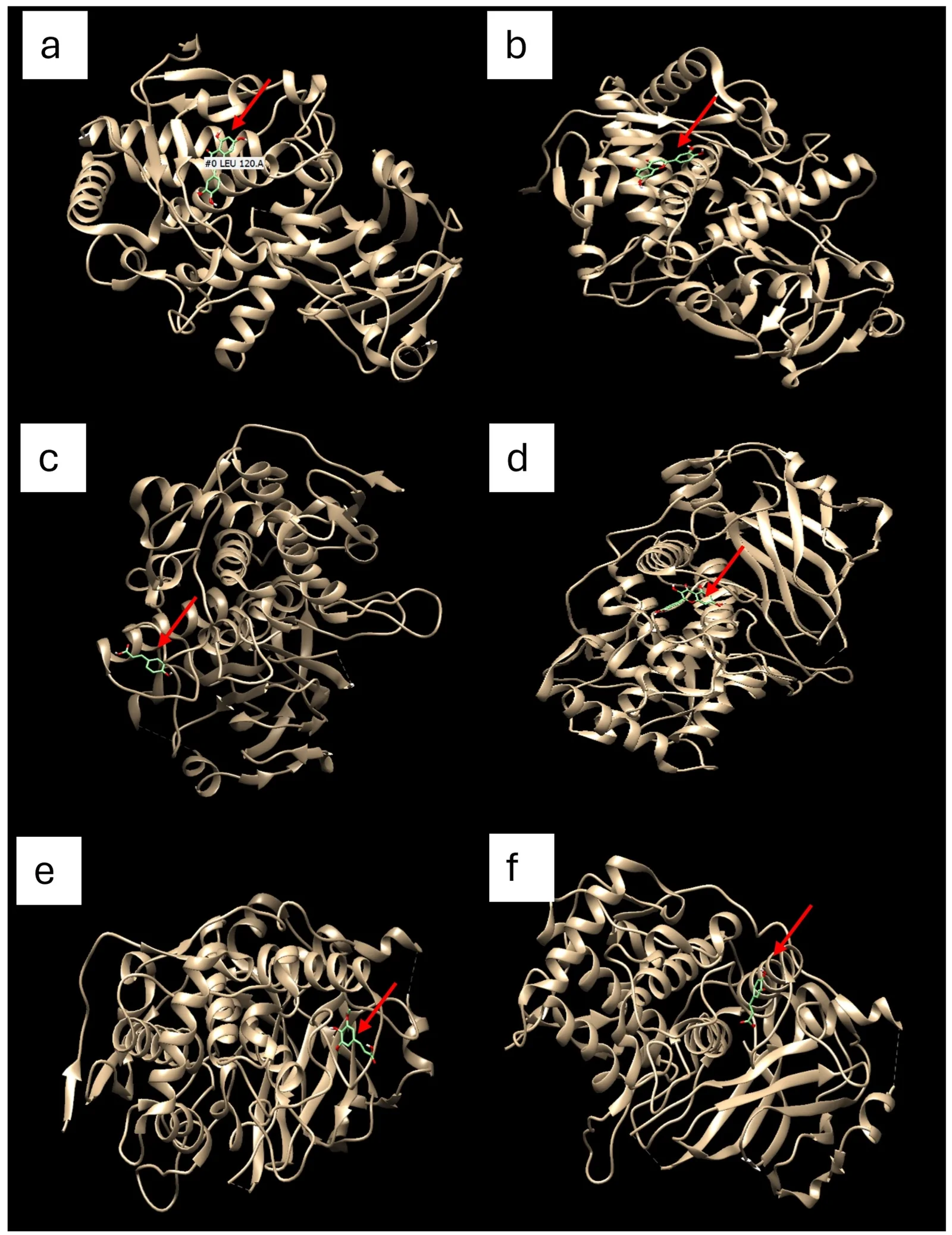
3D structure of molecular docking for different polyphenols against the (MdPPO1) enzyme, gold in colour; the red arrow indicates the interaction of the phenolic acids with the enzyme. Where (**a**) apigenin, (**b**) luteolin, (**c**) p-coumaric acid, (**d**) quercetin, (**e**) sinapic acid, and (**f**) trans-ferulic acid.

Molecular docking analysis was used to evaluate the binding affinities and interactions of polyphenols with MdPPO1, the enzyme responsible for apple browning. Table 5 shows the binding energies of each polyphenol quantified in this study and their interactions with the various amino acids in MdPPO1. Figure 5 demonstrates the 3D structure of the molecular docking between the polyphenols and the amino acids found in the active site of MdPPO1. Table 5 indicates that quercetin obtained the strongest binding energy, −7.8 kcal/mol. Followed by apigenin and luteolin, both displaying binding energies of −7.7 kcal/mol. While p-coumaric, sinapic acid and trans-ferulic acid obtained weaker binding energy, which were −6.2, −6.0 and −5.8 kcal/mol, respectively. These results suggest that the binding affinity between the flavonoids quercetin, apigenin, and luteolin with the receptor protein is much higher compared to the ligands p-coumaric, sinapic acid and trans-ferulic acid. This is because flavonoids contain multiple hydroxyl groups that bind to the protein’s active sites, forming strong hydrogen bonds [52]. This suggests that a microcapsule containing higher flavonoid content would have the strongest affinity for PPO and could potentially exert the strongest anti-browning activity. This is also evident in Table 1: the microcapsules SCG + GA and SCG + GA + MA, with flavonoid contents of 26.01 and 22.11 mg QE/g, resulted in the largest decreases in BI values over 5 days. Compared to the SCG + MA microcapsule, with a flavonoid content of 16.91 mgQE/g.

Figure 5 shows the interactions that occur in the active site of the MdPPO1 enzyme with the different phenolic compounds. These interactions include Pi-sigma, Pi-Pi t-shaped, Pi-alkyl, Pi-anion, Conventional hydrogen bond, and carbon-hydrogen bond, indicating their affinity for the enzyme (Table 5). Among the polyphenols, quercetin, luteolin, and apigenin exhibited various interactions (Pi-sigma, Pi-Pi t-shaped, Pi-alkyl, Pi-anion, and carbon-hydrogen) in addition to multiple conventional hydrogen-bonding interactions, thereby stabilizing them. This demonstrates why quercetin, luteolin, and apigenin exhibited higher binding affinity, as observed in Table 5. On the contrary, p-coumaric and trans-ferulic acids showed a low number of interactions with the PPO enzyme, including carbon-hydrogen and conventional hydrogen bonding, with fewer interactions of Pi-alkyl, alkyl, and Pi anion. More specifically, trans-ferulic acid depicts only two interactions: conventional hydrogen bonding. This explains the lower binding affinity observed for these molecules relative to flavonoids. These results demonstrate that, in application, the flavonoids are likely to be the compounds that contribute to the anti-browning activity of the enzymatic browning of apple juice.

Table 5 demonstrates the binding energies and interactions of polyphenols found in SCG with the apple polyphenol oxidase (MdPPO1) enzyme.

## 4. Conclusions

The study demonstrates the successful microencapsulation of SCG extracts using MA, GA, and a GA + MA combination, yielding a high %EE exceeding 70%. Characterization assays confirmed that maltodextrin and gum arabic can effectively encapsulate the polyphenols present in the SCG crude extract. Molecular docking results show that flavonoids exhibited higher binding affinities than phenolic acids, suggesting that flavonoids are the primary compounds that interact effectively with the PPO enzyme and may act as anti-browning agents. When applied to the juice, microencapsulated SCG extract samples exhibited a decrease in BI compared to the positive and negative controls. This confirms their potential as anti-browning agents. Future studies should focus on extracting purified polyphenols, evaluating their release kinetics from the wall materials, and long-term shelf studies.

## Figures and Tables

**Table 1:** Total phenolic and flavonoid content and antioxidant capacity of the extract before and after encapsulation.

Sample	TPC mg GAE/g	TFC mg QE/g	DPPH %	ABTS %
SCG	37.84 ± 1.24 ^b^	48.47 ± 1.10 ^d^	87.60 ± 0.22 ^b^	84.66 ± 0.66 ^b^
SCG + GA	24.07 ± 2.65 ^a^	26.0 1 ± 1.42 ^c^	83.70 ± 0.91 ^ab^	80.88 ± 0.22 ^b^
SCG + MA	20.36 ± 3.07 ^a^	16.91 ± 0.32 ^a^	79.09 ± 6.03 ^a^	72.84 ± 7.55 ^a^
SCG + MA + GA	23.12 ± 0.44 ^a^	22.11 ± 0.93 ^b^	85.52 ± 1.04 ^b^	83.72 ± 1.31 ^b^

Note: The values in this table are presented as mean ± standard deviation (*n* = 3) and means with the same superscript letters in the same column are significantly different (*p* < 0.05). Abbreviations: TPC (Total phenolic content), TFC (Total flavonoid content), DPPH (2,2-diphenyl-1-picrylhydrazyl), and ABTS (3-ethylbenzothiazoline-6-sulfonic acid). The SCG + MA + GA (SCG extract coated with gum arabic + maltodextrin ), SCG + MA (SCG extract coated with maltodextrin) and SCG + GA (SCG coated with gum arabic).

**Table 2:** Targeted phenolic acid and flavonoid concentrations in SCG.

Phytochemical	Concentration in mg/g
Sinapic acid	4.24 ± 0.18
P-coumaric	0.60 ± 0.01
Luteolin	1.46 ± 0.04
Trans-ferulic acid	14.50 ± 0.06
Quercetin	1.67 ± 0.05
Apigenin	1.11 ± 0.01

The composition of phenolic acids and flavonoids in SCG results is presented as mean ± standard deviation, where *n* = 3.

**Table 3:** Phenolic content (TPC and TFC) and antioxidant capacity (DPPH and ABTS radical scavenging) of apple juice samples with the application of microcapsules and ascorbic acid.

Sample	TPC mg GAE/g	TFC mg QE/g	DPPH %	ABTS %
Positive control	592.12 ± 84.30 ^b^	275.22 ± 20.10 ^c^	54.95 ± 3.28 ^a^	93. 80 ± 0.33 ^d^
Negative control	232.21 ± 28.30 ^a^	138.69 ± 25.26 ^a^	50.19 ± 1.79 ^a^	91.46 ± 0.13 ^c^
Apple juice: MA + GA	288.81 ± 15.83 ^a^	206.74 ± 17.94 ^b^	53.92 ± 3.18 ^a^	84.67 ± 1.18 ^a^
Apple juice: MA	263.99 ± 23.07 ^a^	190.31 ± 8.42 ^b^	58. 81 ± 7.09 ^a^	84.39 ± 0.26 ^a^
Apple juice: GA	297.22 ± 42.79 ^a^	180.19 ± 17.88 ^b^	72.19 ± 6.06 ^b^	88.08 ± 0.60 ^b^

Results are presented as mean ± standard deviation, where *n* = 3 and values with the same superscript letter in the same column are not significantly different from each other (*p* < 0.05). Positive control (apple juice +ascorbic acid) and negative control (apple juice only). Apple juice: MA + GA (apple juice with gum arabic + maltodextrin microcapsule), apple juice: MA (apple juice with maltodextrin microcapsule) and apple juice: GA (apple juice with gum arabic microcapsule).

**Table 4:** Colour parameters and BI of the apple juice sample with the microencapsulated samples and ascorbic acid on day 0 and day 5.

Sample	Day 0				Day 5			
	L*	a*	b*	BI	L*	a*	b*	BI
Negative control	86.91 ± 0.10 ^d^	−1.25 ± 0.03 ^a^	11.52 ± 0.28 ^a^	12.81 ± 0.39 ^a^	86.59 ± 0.31 ^d^	−0.26 ± 0.91 ^b^	13.80 ± 0.27 ^b^	16.72 ± 0.45 ^b^
Positive control	87.46 ± 0.16 ^e^	−1.38 ± 0.19 ^a^	12.45 ± 0.72 ^a^	13.81 ± 1.06 ^a^	88.03 ± 0.41 ^e^	−0.9 ± 0.17 ^a^	8.97 ± 0.33 ^a^	9.67 ± 0.42 ^a^
Apple juice: MA + GA	77.37 ± 0.23 ^a^	1.41 ± 0.06 ^c^	22.77 ± 0.50 ^d^	35.38 ± 0.92 ^c^	78.63 ± 0.39 ^a^	1.68 ± 0.10 ^d^	19.77 ± 0.42 ^d^	29.91 ± 0.79 ^d^
Apple juice: MA	81.22 ± 0.22 ^b^	−0.41 ± 0.02 ^b^	17.85 ± 0.49 ^b^	23.85 ± 0.81 ^b^	81.42 ± 0.33 ^b^	0.44 ± 0.08 ^c^	15.80 ± 0.57 ^c^	21.48 ± 0.89 ^c^
Apple juice: GA	81.83 ± 0.45 ^c^	−0.37 ± 0.07 ^b^	18.94 ± 0.33 ^c^	25.35 ± 0.66 ^b^	82.24 ± 0.21 ^c^	0.51 ± 0.11 ^c^	16.56 ± 0.52 ^c^	22.43 ± 0.91 ^c^

Results are presented as mean ± standard deviation, where *n* = 3, and values with the same superscript letter in the same column are significantly different from each other (*p* < 0.05). Positive control (apple juice + ascorbic acid) and negative control (apple juice). The apple juice: MA + GA (apple juice with gum arabic + maltodextrin microcapsule), Apple juice: MA (apple juice with maltodextrin microcapsule) and apple juice: GA (apple juice with gum arabic microcapsule). L* = lightness, a* = green-red and b* = yellow-blue.

**Table 5:** Table showing binding energy and the interactions of polyphenols found in SCG with the MdPPO1 enzyme.

Ligand	Binding Energy (kcal/mol)	Interacting Amino Acids	Type of Interaction
Quercetin	−7.8	THR A:175, VAL A:188, TRP A:244, THR A:184, HIS A:171 and HIS A:186	Pi-anion, Pi-Pi shaped, Conventional hydrogen bond and carbon-hydrogen bond
Apigenin	−7.7	VAL A:332, ASP A:332, ILE A:231, SER A:230, VAL A:232, TYR A:329 and ARG A:277	Pi-sigma, Pi-alkyl, Pi-anion, Conventional hydrogen bond and carbon-hydrogen bond
Luteolin	−7.7	ARG A:277, VAL A:332, ASP A:331, ILE A:281, THR A:322, VAL A:329 and TYR A:329	Pi-anion, Pi-alkyl, Conventional hydrogen bond and carbon-hydrogen bond
P-coumaric	−6.2	LYS A:190, GLU A:185, and ASP A:186	Pi-anion and Conventional hydrogen bond
Sinapic acid	−6.0	ASP A:186, LYS A:190, VAL A:188, TYR A:181, LEU A:176, TRP A:244, HIS A:171 and THR A:184	Alkyl, Pi-Alkyl, Conventional hydrogen bond and carbon-hydrogen bond
Trans-ferulic acid	−5.8	TRP A:244, ASP A:178 and ASN A:187	Conventional hydrogen bond and carbon-hydrogen bond

Note: The abbreviations under interacting amino acids indicate the following amino acids: THR (Threonine), VAL (Valine), TRP (Tryptophan), HIS (Histidine), ASP (Aspartic acid), ILE (Isoleucine), SER (Serine), TYR (Tyrosine), ARG (Arginine), LYS (Lysine), LEU (Leucine), GLU (Glutamic acid), and ASN (Asparagine).

## Data Availability

The original contributions presented in this study are included in the article. Further inquiries can be directed to the corresponding author.
